# The role of a virtual avatar in attention and memory tasks in Rett syndrome

**DOI:** 10.1186/s12883-021-02212-w

**Published:** 2021-06-14

**Authors:** Rosa Angela Fabio, Giorgia Pergolizzi, Andrea Nucita, Giancarlo Iannizzotto, Tindara Caprì

**Affiliations:** 1 Department of Experimental and Clinical Medicine, University of Messina, Via Bivona, 98122 Messina, Italy; 2 Department of Cognitive Sciences, Psychological, Educational and Cultural Studies, University of Messina, Via Concezione, 6, 98122 Messina, Italy

**Keywords:** Rett syndrome, Avatar, Attention, Memory, Over-selectivity

## Abstract

**Background:**

Since subjects with Rett syndrome (RTT) focus their attention mainly on the faces of people with whom they interact, in this study the role of a human-like smart interactive agent (an avatar) in enhancing cognitive processes is examined. More in depth, this study aimed to understand if, and to what extent, the use of an avatar can improve attention and memory abilities in subjects with RTT.

**Method:**

Thirty-six subjects with RTT participated in the study. All participants performed over-selectivity and memory tasks, for a total of six trials. These trials were randomly presented in two different conditions: with and without virtual avatar.

**Results:**

The results indicated that the participants improved their attention and memory abilities when they performed the tasks with the avatar. There were no improvements when they performed the tasks without the avatar.

**Discussion:**

The results were discussed considering the relationship between motivation, attention and memory in RTT.

**Supplementary Information:**

The online version contains supplementary material available at 10.1186/s12883-021-02212-w.

## Background

Rett syndrome (RTT) is a complex genetic neurological disorder, caused by mutations in the X-linked gene encoding for a regulator of epigenetic gene expression, methyl CpG binding protein (MeCP2). The clinical picture of RTT is defined by loss of hand use and language, with the development of gait abnormalities and hand stereotypies [1–4]. Thus, subjects with RTT often use their eyes to communicate [5–7]. Neurological abnormalities in RTT are reflected in several behavioral and cognitive impairments such as stereotypies, loss of speech and hand skills, gait apraxia, irregular breathing with hyperventilation while awake, and frequent seizures [1–4]. Clinical features of RTT suggest that this disorder is the result of a primary disturbance of neuronal development, resulting in maturation arrest in selected brain regions [8]. For example, this maturation arrest could be caused by defective neurotransmitter systems that fail to provide normal trophic factors [8].

New promising approaches have recently shown that technology-aided programs for subjects with severe/profound and multiple disabilities are useful in enabling performance of daily tasks and improving cognitive abilities [9]. Technology-aided programs together with cognitive rehabilitation can enhance neuroplasticity both with neuropsychological and neurophysiological parameters. Fabio et al. (2016) [10] investigated modifications in behavior and brain activity of patients with RTT by using eye-tracking technology during a discrimination task, while neurophysiological parameters were measured using quantitative EEG (QEEG) analysis. This study showed that patients with RTT become attentive and vigilant to the task and look at the target more quickly and for a longer time. This result was supported by QEEG analysis, that revealed a decrease of beta activity and an increase of right asymmetry. Hence, the study evidenced a positive effect of the application of eye-tracking technology to cognitive rehabilitation on behavioral and neurophysiological parameters in RTT.

Spaghero [9], in a study exploring virtual reality, demonstrated that subjects with intellectual disabilities can present a marked improvement in attention when interacting with virtual objects and events. Therefore, this new approach has been recognized, as it provides a safe environment in which any individual can enjoy pleasurable activities, music [9] and communicating with the family [11]. Another major advantage in using technology-aided interventions is the possibility to acquire huge amounts of data, essential for advanced data analysis such as predictive analysis [12].

A new trend in current technologies is the presentation of a sequence of tasks within a environment, with an avatar giving positive feedback to the subjects on the correct completion of a task, thereby improving motivation [13–16]. Avatars, in the form of graphical characters, able to show human-like facial expressions and gestures, to speak and even react to users’ interaction by interpreting gestures, emotional states and speech, have been exploited for several applications in the past [17]. Furthermore, virtual environments have often been combined with advanced human-computer interaction (HCI) technologies to facilitate user interaction in complex tasks [18].

The use of avatars has been shown to be effective in increasing attention in various disabilities [19–21], however, it has not yet been explored for people with RTT. For example, a study [22] investigated the emotion recognition ability in subjects with autism using an avatar able to recreate emotional facial expressions. Further evidence was provided by experimenting with an avatar in Attention Deficit/Hyperactivity Disorder (ADHD) [23–28]. These studies suggest that an avatar can be used as a computerized pedagogical agent with realistic characteristics; for example, it can appear on a computer screen and guide users through multimedia learning environments, designed to support learning and direct attention to relevant topics. In the study [23], a virtual avatar similar to a young male nerd was created for teaching ADHD subjects, providing options for solving mathematic and logic problems. Results of this study suggested that it could be possible to elicit greater attention in subjects with ADHD and highlight how the presence of a pedagogical agent may improve the performance of subjects.

The role of a computerized pedagogical agent is also supported by other theories. According to Computers Are Social Actors (CASA), for example, people respond to the media in the same way they would respond to humans, so the avatar would become a real social model [29, 30]. Wang, Wenjing and Heping [31] also states that people learn better from multimedia presentations in the presence of a pedagogical agent, recognizing social presence. For these reasons, the use of the avatar becomes effective for students to structure the appropriate processes and improve attention and learning [32] by strengthening their interests [33].

Other evidence on the role of the virtual avatar were provided in a study [21] in which subjects with ADHD underwent three experimental conditions. In the first condition, the avatar simply provided instructions; in the second condition the avatar presented the instructions and also provided feedback on the student’s attention; in the third condition the avatar was not presented. Results showed that the presence of the virtual avatar increased the problem-solving ability of the subjects. These benefits were also confirmed in the work of Shema-Shiratzky, Brozgol CornejoThumm [34]. Summarizing, avatars showing behaviours that the users feel as familiar [35] can become real tutors, coaches, motivators, mentors, models to emulate [36].

This study aims to examine whether the use of an avatar can improve attention and memory abilities in subjects with RTT. The underlying logic is that the motivation process significantly affects some cognitive functions, such as attention and memory. Since subjects with RTT are very motivated to look at the face of therapists and parents, an avatar acting human-like gestures, speech, gaze and behaviors may be very motivating to join in the learning process.

Attention and memory tasks were presented with two conditions through eye tracking technology. In the first condition, the avatar gave the instructions and asked the patient to give the answer. In the second condition there was no avatar. A voice gave the instruction and solicited the replies. The hypothesis was open: on one hand it may be that the avatar, acting as a social motivational model [30] can induce subjects with RTT to focus and memorize more efficiently; on the other hand, based on the redundancy theory of Mayer [29], since the avatar is a third element it may be that it acts like a distractor and, not necessarily improve attention and memory.

Furthermore, since the eye tracking methodology helps to distinguish the encoding phase (with the registration of the length of fixations) and the retrieval phases (with the number of items recalled), in this study we want to understand if the avatar is effective in the encoding phase or in the retrieval phase or both. As shown in previous studies with ADHD children[21, 23] we expect that, if the subjects with RTT show a high level of length of fixation in the condition with the avatar, such a condition will produce benefits also in the retrieval phase.

## Method

### Participants

Forty-one subjects with a diagnosis of RTT took part in the experiment. Forty were female and one was male. Their families had been contacted by the Italian association for Rett syndrome (AIRETT) that asked them to participate in the study. The families come from all over Italy. Five subjects were excluded from the study since they were not able to focus on the stimuli of the monitor. For this reason, finally, 36 subjects participated in the study. They ranged in age between 4 and 36 years. A general assessment was carried out by a psychologist through the Vineland Adaptive Behaviour Scale (VABS)[37] and the Rett Syndrome Rating Scale (RARS) [38]. Thirty-one subjects and one male attended schools or socio-educational centres; four subjects were assisted by an educator at home. All showed little or no purposeful hand use and pervasive hand stereotypies were striking. Ambulation was preserved in 19 subjects. Table 1 shows the chronological age of the participants, the RARS scores as well as the VABS Scores.

**Table 1:** Descriptive characteristics of the patients with Rett Syndrome

Clinical stage	Age	MeCP2 Mutation	RARS^1^ scores	Level of^2^ severity	VABS^3^ scores
II	4	R306C	66.5	Moderate	83
II	4	R255X	80.5	severe	40
II	4	PT158M	52.5	Moderate	60
II	4	R306C	74	Moderate	50
II	4	/	71.5	Moderate	56
III	5	R294X	66.5	Moderate	80
III	5	P322A	58.5	Moderate	75
III	7	/	47.5	Moderate	60
III	7	R168X	51.5	Moderate	82
III	8	/	48.5	Mild	121
III	8	R133C	35	Mild	140
III	10	FOXG1	86.5	severe	35
III	10	T158M	81	Severe	33
III	11	R255X	66	Moderate	83
III	12	R306C	69	Moderate	88
III	12	R306C	59	Moderate	84
III	13	S355fsX37	63.50	Moderate	110
III	14	/	56	Moderate	58
III	14	R270X	61.5	Moderate	76
III	14	P322A	52.5	Moderate	86
III	16	T158M	71	Moderate	92
III	16	168RX	62	Moderate	131
III	16	R133C	64.5	Moderate	110
III	17	arg168stop	37	Mild	121
III	18	/	67.5	Moderate	107
III	19	R255C	74.5	Moderate	84
III	20	CDLK5	61	Moderate	56
III	20	P322L	58.7	Moderate	105
III	22	P389X	66.5	Moderate	84
III	26	R270X	56.5	Moderate	65
IV	29	R255C	72	Moderate	86
IV	29	R135C	39	Mild	168
IV	31	R255C	59.5	Moderate	165
IV	35	/	47	Mild	118
IV	36	T158M	59	Moderate	63

^1^Rett Assessment Rating Scales (RARS)

^2^Levels of severity according to the total scores of RARS (Mild = 0–55; Moderate = 56–81; Severe= > 81)

^3^Vineland Adaptive Behaviour Scales-Interview second edition (VABS)

### Material

A Tobii Series-I eye-tracker was used to record the participants’ visual scanning. This tool records the following ocular movements: the location and duration of ocular fixations (pause of eye movement on an object of interest) and saccadic movements (rapid movements between fixations). Passive gaze tracing (LC Technologies, Sao Paulo, Brazil) software was used to generate gaze data during visuals scanning. It was possible to choice the areas of interest (AOI) within the images chosen for the statistical analysis of eye tracking. An AOI cluster can be defined as selected specific areas that are used for both attention and recalling details of the images.

The eye-tracker was used for both the overselectivity paradigm and the memory paradigm. The avatar was created using an educational platform “Voki for Education” (https://www.voki.com/). Voki is a free collection of customizable speaking avatars for teachers and educators that allows users to create a precise profile of a talking character. Voki is created by Oddcast and can be customized to look like humans, cartoons, and/or animals.

The characteristics selected for the creation of the avatar were chosen through a pre-calibration, carried out during the 2018 Airett Campus in which several subjects with RTT spent their holidays with family and educators. The pre-calibration was fundamental as it allowed to include the avatar that the RTT subjects prefer. Following, the materials of both paradigms will be presented.

### Memory paradigm

The memory test was implemented. The story-cartoon presented with Tobii eye-tracker was easy to understand and remember, and the descriptions of facts were presented in a logical order. Four cartoon sequences were extracted, two from the “ant Bibi” and two from the “Pimpa on the beach” animation movies, respectively. They were chosen out of seven cartoon sequences presented to 31 3-year-old children and calibrated based on their comprehension of the story (> 90%) and on their recalling indices (> 90%). The questions have various levels of difficulty, from simple recognition of the main character of the story, to recognition of the emotional states of the characters, to identification of the actions within the story.

Each cartoon sequence contained 8 significant memory indices (Table 2). Both cartoon sequences “Ant Bibi” and “Pimpa on the Beach” lasted 2:30 min.

**Table 2:** Significant indexes of cartoon sequence

Ant Bibi	Pimpa on the Beach
Main character	Main character
Secondary character	Action of Pimpa
Colour of main character	Secondary character
Ant character	Action on the beach
Action of Pimpa	Emotions of Pimpa
Underground	Crayfish character
Emotions of Pimpa	Underground
Final action	Final action

The test was carried out for each patient. After each cartoon was presented through the eye-tracker, the participants were asked to perform immediate recall of the cartoon with a recognition test with 8 questions regarding the story (Table 3).

**Table 3:** Questions related to the significant indexes of the cartoon sequence

Ant Bibi-1	Pimpa on the Beach-1
1. Who was the main character of the story?	1. Who was the main character from the story?
2. Who met Pimpa at the beginning of the story?	2. What was Pimpa doing?
3. What colour was Pimpa?	3. Did Armando want to play with Pimpa?
4. Who met Pimpa?	4. Who did Pimpa play with on the beach?
5. What was Pimpa taking the sack or the bucket?	5. Was Pimpa sad or happy?
6. Were there clouds or stars?	6. Who did you meet after the Pimpa?
7. How happy or sad was Pimpa?	7. Were there sun or stars?
8. What did Pimpa do at the end of the story?	8. What did Pimpa do in the end?
Ant Bibi-2	Pimpa on the Beach-2
1. Who was the main character of the story?	1. Who was the main character of the story?
2. What the colour was the sky?	2. What the colour was the Pimpa’s ball?
3. Who met Pimpa?	3. Was there a boat or a car?
4. What Ant Bibi give her?	4. What the colour was the boat?
5. Shoes were clear or unclear?	5. What did the boat do?
6. What ate Pimpa and Ant Bibi?	6. How happy or sad was Pimpa?
7. How happi or sad was Pimpa?	7. Were there sun or stars?
8. What did Pimpa do at the end of the story?	8. What did Pimpa do at the end of the story?

For each of the relevant indexes two cards were presented on the screen, the correct response (CR) and the distractor answer. The scores were 1 or 0: if the response was corrected, 1 score, if the response was the distractor stimulus, 0 score.

### Overselectivity paradigm

**Fig. 1:**
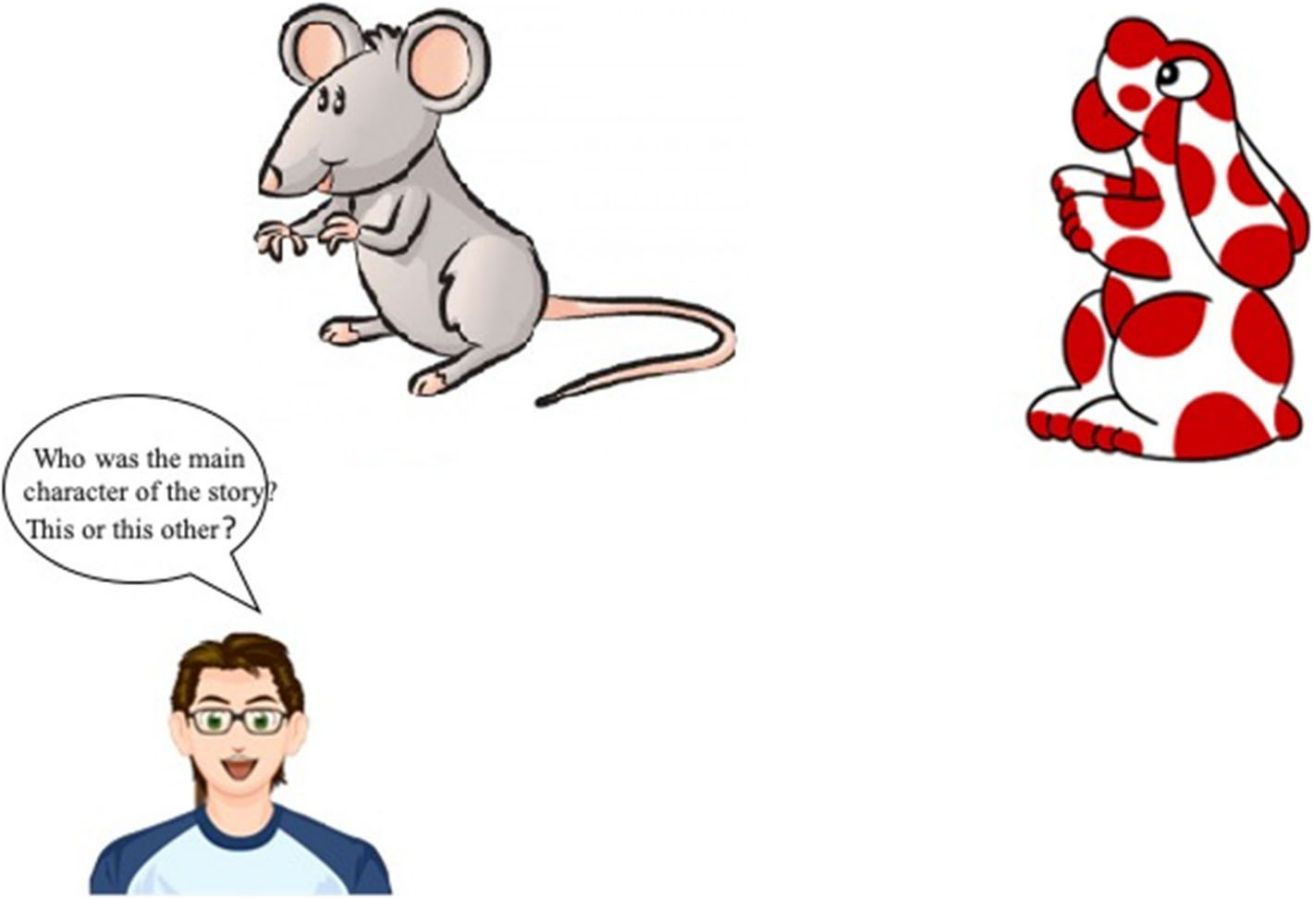
Recognition test: target and distractor. The images in Figs. 1, 2 and 3 were created by two co-authors using Nero PhotoSnap for Windows 10 (https://it.all10soft.com/nero-photosnap-windows-10/). The images are freely available

**Fig. 2:**
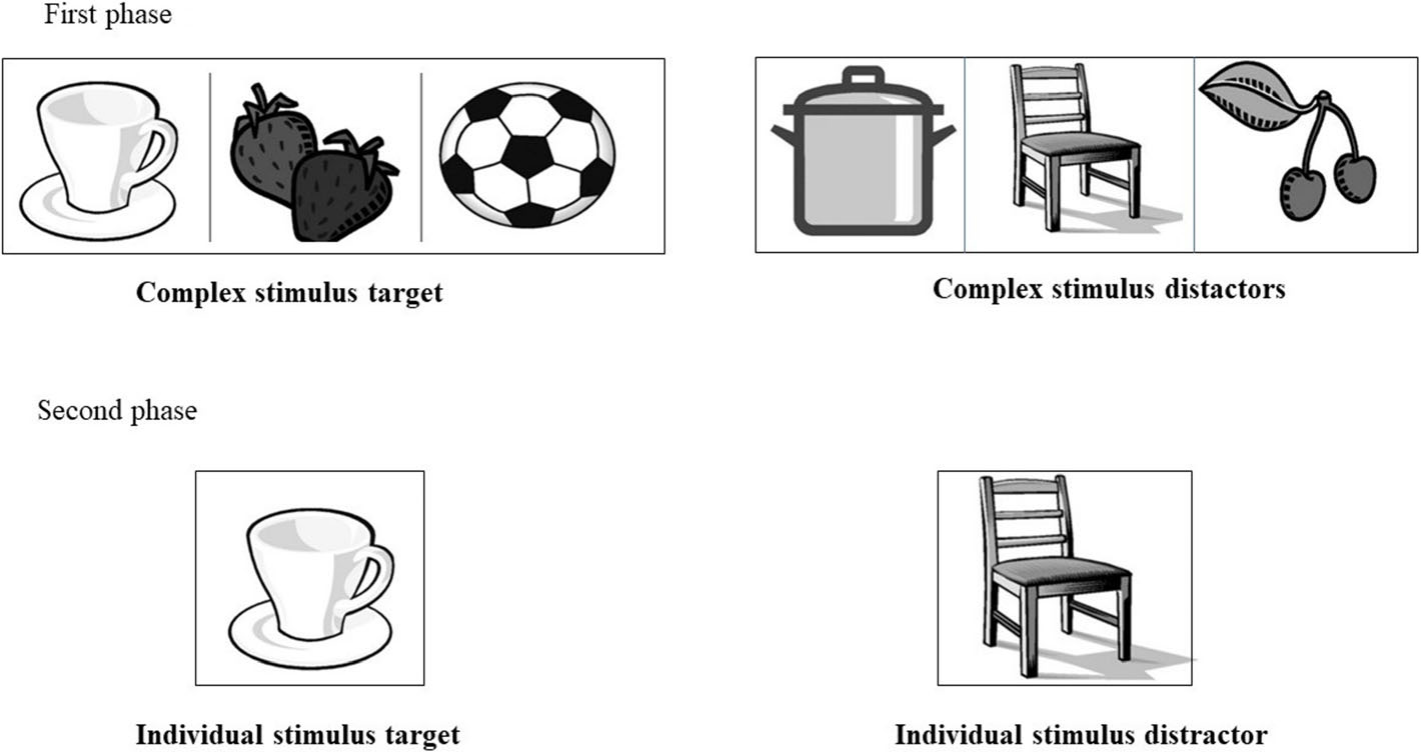
Overselectivity stimuli. The images in Figs. 1, 2 and 3 were created by two co-authors using Nero PhotoSnap for Windows 10 (https://it.all10soft.com/nero-photosnap-windows-10/). The images are freely available

In this paradigm, 2 cards of 10 cm × 30 cm, each one reporting a different complex stimulus composed of three familiar objects shown in black and white, were presented on the screen of the Tobii eye-tracker. In the second phase, individual stimuli, consisting of cards of about 10 cm × 10 cm, were presented on the screen. Each card represented a single familiar object previously included in the target complex stimulus (Fig. 1). The cards were calibrated in a previous study [39]. In the condition with virtual avatar, the avatar presented both the complex stimuli and the individual stimuli between which the participant had to choose (Fig. 2, phase 1 and 2). In the condition without virtual avatar, no avatar was presented to participants.

**Fig. 3:**
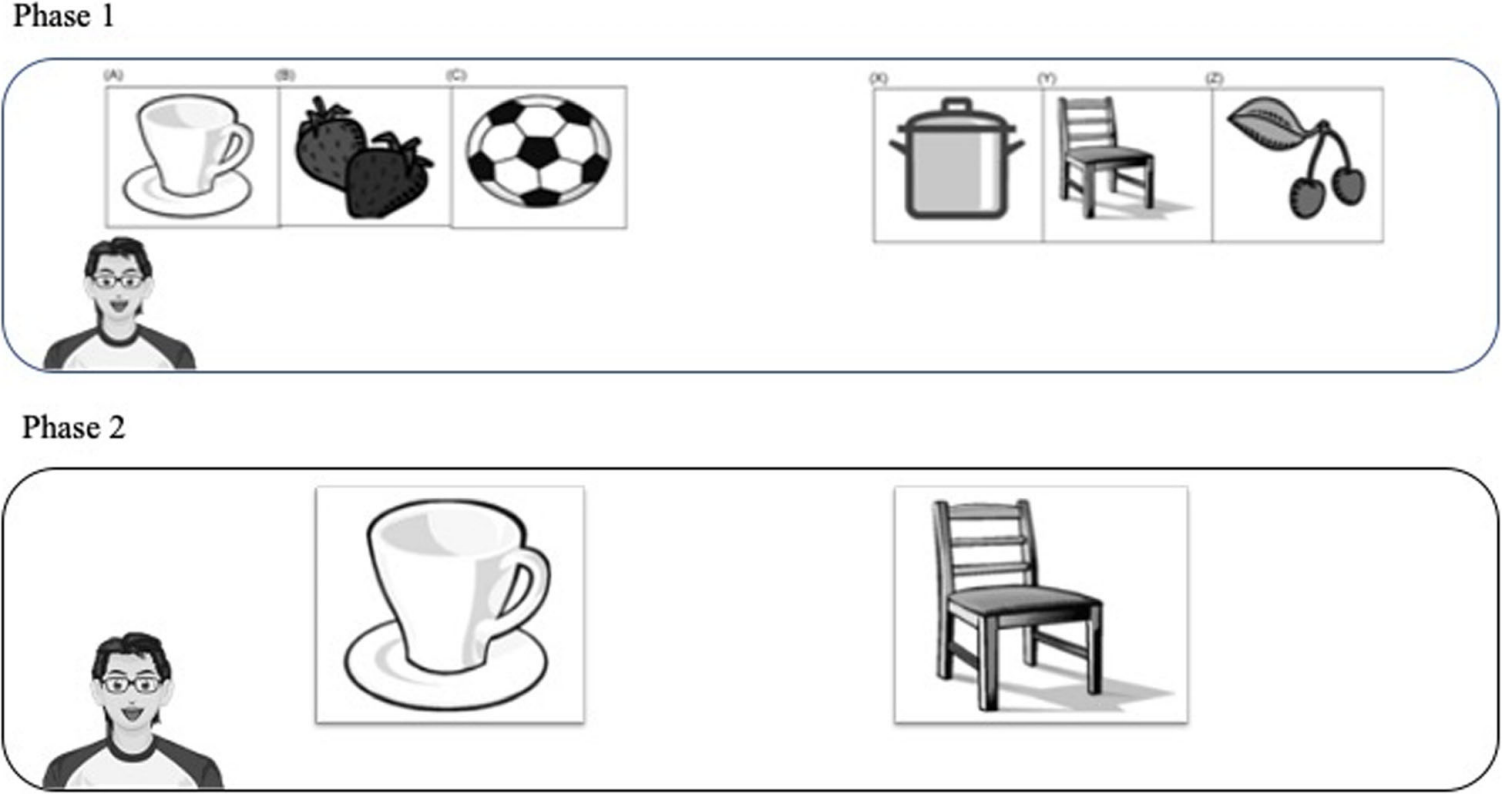
Recognition test in overselectivity paradigm with the avatar. The images in Figs. 1, 2 and 3 were created by two co-authors using Nero PhotoSnap for Windows 10 (https://it.all10soft.com/nero-photosnap-windows-10/). The images are freely available

### Vineland adaptive behavior scales-interview second edition (VABS)

The VABS is subdivided into four domains: communication, daily living, socialization and motor skills*.* The interviewer asks general questions pertaining to the patient’s functioning in each domain and uses the responses to rate the examinee on each critical behaviour item (2: always present, 1: sometimes present, 0: seldom or never present). Typical interviews require approximately 1 h. A total score is computed by summing the individual ratings for each scale. The reliability of VABS was established as follows: split-half, 0.73–0.93 for the communication domain, 0.83–0.92 for daily living skills, 0.78–0.94 for socialization, 0.70–0.95 for motor skills, 0.84–0.98 for adaptive behaviour composite, 0.77–0.88 for maladaptive behaviour. The interrupter reliability coefficients for the survey and expanded forms ranged from 0.62 to 0.75. Standard error of measurement ranged from 3.4 to 8.2 over the four domains, and from 2.2 to 4.9 for the Adaptive Behaviour Composite, on the survey form. The VABS was obtained for use in this study because it was bought by the University of Messina.

### Rett assessment rating scales (RARS)

The RARS is a standardized scale used to evaluate patients with RTT. It is organized into seven domains: cognitive, sensorial, motor, emotional, autonomy, typical characteristics of the disease and of behaviour. Typical characteristics of the disease and behaviour domains measure the following characteristics: mood swings, convulsions, dyspnoea, hyperactivity, anxiety, aggressivity, bruxism, oculogyric crises, epilepsy, aerophagia, muscle tension and food preferences. A total of 31 items was generated as representative of the profile of RTT. Each item is provided with a brief glossary explaining its meaning in a few words. Each item is rated on a 4-point scale, where 1 = within normal limits, 2 = infrequent or low abnormality, 3 = frequent or medium-high abnormality, and 4 = strong abnormality. Intermediate ratings are possible, for example, an answer between 2 and 3 points is rated as 2.5. For each item, the evaluator circles the number corresponding to the best description of the patient. After a patient has been rated on all 31 items, a total score is computed by summing the individual ratings. This total score allows the evaluator to identify the level of severity of RTT, conceptualized as a continuum ranging from mild symptoms to severe deficits (Mild = 0–55; Moderate = 56–81; Severe= > 81). The RARS was established by a standardization procedure, involving a sample of 220 patients with RTT, proving that the instrument is statistically valid and reliable. Skewness and kurtosis values, calculated for the distribution of the total score, are 0.110 and 0.352, respectively. Distribution is found to be normal. Cronbach’s alpha is used to determine the internal consistency for the whole scale and sub-scales. Total alpha is 0.912, and the internal consistency of the sub-scales is high (from 0.811 to 0.934).

### Procedure

The experiment was carried out in a quiet room during the 2019 Rett summer campus of the AIRETT. The examiner administered the VABS and the RARS through an interview with the parents of the subjects with RTT and the educators. Participants sat in a dimly lit room of the association in front of the eye-tracker screen at 30 cm and the direction of the gaze was determined according to the Pupil Centre/Corneal Reflection Method in low-intensity infrared light.

The eye tracker was positioned in such a way that ambient lighting did not affect the recordings. The eye tracking equipment was calibrated for each participant at the beginning of the experiment. Gaze fixations of at least 1000 ms within a region of 2°– 3° around each calibration point were considered accurate.

The two tasks of this experiment were presented randomly in two conditions with and without avatar, as given in Tables 4 and 5. All participants performed over-selectivity and memory tasks. There were six trials: four different stories-cartoon of “Pimpa” in the memory task, and two over-selectivity trials with different familiar stimuli. These trials were randomly presented in two conditions (with and without virtual avatar). The experiment lasted 30 min in total.

**Table 4:** Memory task procedure

Experimental condition	Procedure
With avatar	The avatar appeared initially on the whole screen of the Tobii I-15 and said “Hi, my name is Giorgio. Watch this cartoon with me!” Then the avatar became smaller and moved to the lower left part of the screen. During the cartoon, it only moves its eyes and head in a stereotyped way to make the avatar seem alive. After watching the first cartoon, the avatar appeared again and says “Hello, we will play together now!” Then the avatar started by asking the participant the 8 questions. Participants had to choose the correct answer with their eyes and avoid focusing on the distractor. Then the avatar appeared again and repeated the process with a second video.
Without avatar	No avatar was presented to participants. The story-cartoon was presented with Tobii eye-tracker.

**Table 5:** Overselectivity paradigm procedure

Experimental condition	
Without avatar	In the first phase, two images placed 40 cm from each other, reporting complex stimuli (ABC, correct stimulus; XYZ, incorrect stimulus (Fig. 2) were presented. The examiner presented each subject with the correct complex stimulus described as the “correct one”; both the correct and incorrect cards were then presented on the screen in front of each subject who was subsequently asked: “Which is the correct one?” Forty-five seconds were allowed to answer the question. The subjects could answer by grasping an image or by looking at it. If the subject chose the correct card (ABC) during the 45 s, the examiner gave them a verbal reinforcement (e.g. “Great!” “Very good!”). If the subject chose the incorrect image (XYZ) or did not choose any image during 45 s, both were removed, and the ‘no’ answer was coded. A new possibility of choice started after 10 s. In the second phase, the examiner used the cards reporting individual objects extracted both by correct and incorrect complex stimuli by devising 9 different pairs of individual stimuli from the combination of A with Y, B with X etc. The examiner asked every participant to choose the correct stimulus.
With avatar	The avatar presented both the complex stimuli and the individual stimuli between which the participant had to choose (phase 1 and 2).

### Measures

Memory task: fixation length (FL) of the correct stimuli related to the significant memory indices during the vision of the cartoon and the number of the recalled correct indexes. FL refers to the amount of time (seconds) spent by the subject when looking at the correct stimulus. Total fixation length refers to the sum of the time spent in looking at each significant index during the vision of the cartoon. Fixations were extracted using a threshold of 100 ms.

Overselectivity task: FL of the complex correct stimulus and the number of the individual correct recalled images.

### Statistical analyses

The data were analysed using SPSS version 22.0 for Mac. The descriptive statistics of the dependent variables were tabulated and examined. Alpha level was set to 0.05 for all statistical tests. In the case of significant effects, the effect size of the test was reported. The relationship between continuous variables was evaluated by determining Pearson’s r; group comparisons were conducted using t-test for paired samples.

## Results

With reference to the memory paradigm, a preliminary analysis showed that the two cartoons “Ant Bibi” and “Pimpa on the Beach” showed no statistical differences either with reference to fixation length of the total correct stimuli, t (35)=1.1, *p* = .43, or the number of the correct recalled indexes, t (35)=0.76, *p* = .67; for this reason in the following statistical analysis the mean of indexes of both cartoons is used. Table 6 shows means and standard deviations of the two conditions.

**Table 6:** Means (M) and Standard deviations (SD) of the parameters of the memory task

Conditions	Parameters	M (SD)
With Avatar	fixation length of the total correct stimuli	26.37 (12.95)
	number of the recalled correct indexes	5.23 (1.10)
Without Avatar	fixation length of the total correct stimuli	15.95 (11.82)
	number of the recalled correct indexes	3.23 (1.99)

With reference to the length of the total correct stimuli results show significant differences, t (35) = 4.55, *p* < .0001. This means that the time spent in looking at each significant index during the vision of the cartoon was higher when the avatar helped the subjects than when the avatar was not present. With reference to the number of the recalled correct indexes, results show significant differences, t (35) = 6.02, *p* < .0001. Subjects with RTT recall more significant indexes when the avatar is present than when it is absent.

With reference to the overselectivity paradigm, FL of the complex correct stimulus show significant differences, t (35) = 6.61, *p* < .001. This means that the time spent in looking at the correct complex stimulus was higher when the avatar was present than when the avatar was not present. With reference to the number of the individual correct recalled images, results show significant differences, t (35) = 7.01, *p* < .0001. Subjects with RTT recall more significant indexes when the avatar is present than when it is absent. Table 7 shows means and standard deviations of the parameters of the overselectivity task in the two conditions.

**Table 7:** Means (M) and Standard deviations (SD) of the parameters of the overselectivity task

Conditions	Parameters	M (SD)
With Avatar	fixation of the complex correct stimuli	28.51 (9.22)
	number of correct individual stimuli recalled	6.19 (2.33)
Without Avatar	fixation of the complex correct stimuli	18.32 (11.01)
	number of the recalled correct indexes	3.72 (0.99)

With reference to the second question here addressed, the avatar helps both encoding and retrieval phases. Pearson’s coefficient r was chosen as the measure of the strength of correlation. With reference to the memory task, the relationship between FL of the correct stimuli and the number of correctly recalled indexes is high, r (35) = .678, *p* < .001. By comparing the level of severity (RARS) with FL and the number of corrected response in the condition with avatar, they showed statistically significant differences correlations, r (35) = − .446, *p* < .001; r (35) = − .548, *p* < .001. No statistically significant correlations between memory parameters, age and VABS were found.

With reference to the overselectivity task, the relationship between FL of the complex correct stimulus and the number of the individual correct recalled images is high, r (35) = .52, *p* < .001. Compared with RARS, FL and CR showed statistically significant correlations in the condition with avatar, r (35) = − .404, *p* < .05; r (35) = − .478, *p* < .001. No statistically significant correlations between overselectivity parameters, age and VABS were found.

In the condition without avatar, no significant correlations between the two tasks, age, RARS and VABS were found.

## Discussion

This study addressed two questions: whether the avatar can act as a social motivational model and improve attention and memory and whether it is redundant acting like a distractor and disrupting attention and memory. Both results, related to the two paradigms here analysed, showed that subjects with RTT had a high level of length of fixation in the condition with the avatar and this produced benefits also in the retrieval phase. In addition, the results of the present study indicated that patients with RTT, when performing the two tasks in the condition with avatar, presented a statistically significant improvement in attention and memory abilities. Moreover, correlational analysis between the level of severity of RTT and the two tasks (memory and overselectivity paradigms) showed significant negative relationships. This indicates that at higher levels of severity, the performance of patients with RTT decrease. Thus, there is a relationship between the clinical status of the patients who participated in the study and the test results.

Taken together, these findings indicate that the avatar acts as a cognitive strengthener. Our results support the role of the avatar in learning environments and in the attention process, as social model. These results are in line with the CASA theory. This theory assumes that people respond to the media in the same way they would respond to humans [29]. Wang’s theory also states that learning can be optimized by the presence of a pedagogical agent on the screen [31]. For these reasons, the use of the avatar becomes effective for subjects with RTT to structure the appropriate processes and improve their attention and their learning [40–46].

In line with this [47–49], our avatar not only gives instructions on the relevant aspects of the coding phase but also helps the subjects to better recall the contents. Hence, our results indicate that an interactive avatar that helps subjects with RTT to better direct their attention can concretely improve cognitive performance.

The limitations of the present study are related to the graphical, behavioural, and technological characteristics of the avatar and the sample size. With reference to the characteristics of the avatar, they can affect the performance of subjects with RTT, so it is important to understand what technological, graphical and behavioural conditions are necessary to improve the performance. With reference to the size of the sample, there may be constraints to the generalizability of the results because the sample size is small. However, given that the effect size is adequate, the results can be considered reliable.

The present work can be considered a preliminary experiment. Future studies are needed to understand the conditions under which an avatar can improve the learning process of subjects with RTT. In addition, future research could consider the correlation found in this study, between the severity level of RTT and the cognitive performance of the subjects, as confounding factors. Furthermore, it could be interesting to replicate this study with neurological measurements, such as brain activity, in order to verify whether the avatar can also act as a social motivational model and improve attention and memory at neurological level.

## Conclusion

We consider the findings reported in this article a relevant contribution to the introduction of a new generation of embodied interactive avatars aimed at supporting subjects with RTT in their everyday activities [50, 51]. These findings can help families and educators to identify what software can be effectively used to help children and girls with RTT, and also software designers to make good evidence-based choices to offer more focused software, capable of giving significant performance improvements [52–60].

## Supplementary Information

**Additional file 1:.**

## Data Availability

We enclosed the full data set as additional supporting file. We had the permission to use the VABS because it was bought by the University of Messina.

## Abbreviations

AOI: Areas of interest
ADHD: Attention Deficit/Hyperactivity Disorder
CASA: Computers Are Social Actors
FL: Fixation length
HCI: Human-computer interaction
AIRETT: Italian association for Rett syndrome
RTT: Rett syndrome
RARS: Rett Syndrome Rating Scale
VABS: Vineland Adaptive Behaviour Scale
